# Pediatric Nonaccidental Trauma: Experience at a Level 1 Trauma Center

**DOI:** 10.1155/2020/6621992

**Published:** 2020-12-29

**Authors:** Taylor Goldman, Sathyaprasad Burjonrappa

**Affiliations:** ^1^Department of Pediatric Surgery, University of South Florida College of Medicine, Florida, USA; ^2^Rutgers State University of New Jersey, 1 Robert Wood Johnson Place, MEB 504, New Brunswick, NJ, 08901 New Jersey, USA

## Abstract

**Introduction:**

Pediatric nonaccidental trauma (NAT) is difficult to diagnose. Several isolated injuries in NAT could happen in the setting of accidental trauma (AT), and having a high index of suspicion is important to correctly identify abuse. NAT has a significant mortality rate if the sentinel event is not adequately diagnosed, and the infant is not separated from the perpetrator. Level 1 pediatric trauma centers (PTC) see a significant number of NAT. We evaluated the injury patterns of NAT admissions at our level 1 PTC.

**Methods:**

Retrospective analysis of all cases of NAT for children under the age of two admitted at an ACS level 1 pediatric trauma center between the years of 2016 and 2018. Charts were queried for demographic data, injury patterns, mortality, and disposition. Correlation between disposition status and injury patterns was performed. The Fisher Exact test and student *t*-test were used to study the significance of differences in categorical and continuous data, respectively.

**Results:**

32/91 (35%) trauma patients under the age of two years were diagnosed as NAT in the three-year study period. 21/32 (39%) male and 11/26 (42%) female admissions were confirmed NAT (*p* = NS). 20 were under 1 year of age, and 12 were aged between 1 and 2 years (*p* = NS). 13 (41%) were Caucasian, 6 (19%) were Hispanic/Latino, 11 (34%) were Black, and 2(6%) were of unknown ethnicity (*p* = NS). Facial, torso, lower extremity, retinal, and internal organ injury were significantly more common with NAT. Medicaid coverage was noted in 31/32 (97%) NAT patients. 20/32 (62.5%) patients were legally displaced as a result of the NAT.

**Conclusion:**

1/3^rd^ of all admissions at a pediatric level 1 trauma center were identified as NAT. A high index of suspicion is necessary to not miss NAT, as injury patterns are variable. Nearly 1/3^rd^ of all victims go back to the same environment where they sustained NAT increasing their susceptibility to future NAT.

## 1. Introduction

In a landmark report, in 2017, the United States Department of Health and Human Services (HHS) found that around 123,000 children were victims of nonaccidental trauma (NAT), and 715 of these children died [1]. Victims of NAT face a significant risk of subsequent events of abuse [2]. In one study, victims who were previously treated for NAT had a 24.5% mortality rate, whereas victims who were only treated once had a 9.9% mortality rate [3]. It is important for the healthcare providers to correctly identify risk factors and injury patterns in NAT so that appropriate authorities can take legal action to remove the child from an unsafe environment in the case of proven abuse. This study seeks to understand the demographics, injury patterns, and disposition patterns following NAT admission at a large urban pediatric level 1 trauma center.

## 2. Methods

The charts of all cases of trauma under the age of 2 years admitted to an American College of Surgeons (ACS) level 1 Pediatric Trauma Center (PTC), over a recent three-year period (2016-2018), were retrospectively reviewed after Institutional Review Board approval. Discharge records were used to categorize the patients as AT (accidental trauma) or NAT. Charts that met the inclusion criteria were queried for demographic data: age, sex, race, insurance coverage, location-based injury pattern, and length of stay. Age was divided into two groups: younger than 1 year and between 1 and 2 years. Classification of the injury patterns seen was broken up into the following groups: skin and soft tissue injury (facial, scalp, upper extremity (UE), torso, and lower extremity (LE)), cerebral, bone fracture(s), retinal, internal organ, and multiple injury patterns.

Categorical variables were studied for statistical significance using the Fisher Exact test. Broad-category injury patterns, such as bone fractures and cerebral injuries, were further analyzed to determine if specific injuries had a higher correlation to NAT. We used the total study population as the base incidence (assuming a normal distribution) to compare the significance of incidence amongst other ethnic groups. The significance of continuous data was evaluated using the student *t*-test. A *p* value of < 0.05 was used to represent significance.

## 3. Results

Ninety-one patients, who met inclusion criteria, were admitted during the study period. Sixty-two patients (68.1%) were under the age of 1 year, and 29 (31.8%) were in the 1-2-year age group. Fifty-four patients (59.3%) were male. Thirty-eight patients (41.8%) were Caucasian, 25 (27.5%) were Hispanic/Latino, 21 (23.7%) were Black, and 7 (7.7%) were other or unknown. Seventy-seven patients (84.6%) had Medicaid coverage, 10 (10.9%) had private payer insurance, and 4 (4.4%) dual coverage. Fifty-nine cases (64.8%) were AT, and 32 (35.2%) were NAT.  Table 1 compares demographic data between AT and NAT groups.

Age, gender, and race were not significantly different between AT and NAT groups. There was a significantly higher proportion of Medicaid coverage in NAT cases: 31 (96.9%) as compared to AT 44 (74.6%) (*p* = 0.03).

Table 2 demonstrates the injury patterns seen in nonaccidental trauma compared to accidental trauma. Skin and soft tissue injuries involving the face, torso, and lower extremity were significantly more common in NAT (*p* < 0.05). Extremity fractures were significantly more common in NAT (*p* < 0.05). Retinal injuries were statistically significant for NAT compared to accidental (Table 3). They were found in 4 (12.5%) of NAT cases and 1 (1.7%) of AT cases (*p* = 0.03). Internal organ injuries were also statistically significant for NAT over accidental injury: they were seen in 5 (15.6%) of NAT cases and 2 (3.4%) of non-NAT cases in the age group studied (*p* = 0.03) (Table 4). There was no significant difference in the incidence or pattern of cerebral injury between AT [32/59 (54.2%)] and NAT [12/39 (37.5%)] groups (Table 5). Multiple injury patterns were seen in 22 (68.8%) of NAT cases and 39 (66.1%) accidental cases (NS).

AT cases were grouped into five distinct categories: fall, motor vehicle crash, pedestrian, animal attack, and underlying neurological disorder (seizure). 44 (74.6%) were caused by fall, 8 (13.6%) were either a passenger or a pedestrian in a motor vehicle collision (MVC), 4 (6.8%) were victims of an animal attack, and 3 (5.1%) were due to an underlying neurological disorder.

The average LOS for AT victims was 3.22 (SD 1.79, range 1-12, median: 3 days). The average LOS for NAT victims was 7.61 (SD 12.7, range 1-71, median: 4 days). 20/32 patients were legally displaced, and 11 were discharged back to their home after Child Protection Services (CPS) evaluation. One child in the NAT group died.

## 4. Discussion

It is important to have a high index of suspicion for NAT amongst trauma admissions in the 0-2-year population. Nearly one-third of trauma admissions to an ACS level I pediatric Trauma Center in this study were secondary to NAT. However, in our experience, none of the common demographic factors can satisfactorily screen and identify NAT victims. In this study, the prevalence of NAT was not significantly different between genders, races, or age groups. While some studies have suggested that NAT may be more common in males, others found that the rate of abuse was higher in African American children. Neither was a significant factor in this study [1, 4–7]. We do concur with other studies that NAT most often occurs in children under the age of 2 [4, 5, 7]. This study and others have identified that Medicaid insurance coverage was higher amongst NAT victims as compared to private insurance [3, 7, 8]. We strongly believe that based on our experience, each and every trauma victim in the 0-2 years age group should be screened for potential NAT. This can be done with minimal impact on length of stay and cost but significant improvement in safety of discharge.

Injury pattern screening may offer clues to the etiology of the trauma. We found a significantly higher incidence of skin and soft tissue injuries to the face, torso, and lower extremity, fractures to the extremity, retinal injuries, and internal organ injuries in NAT as compared to AT victims.

It is pertinent to screen children with skin and soft tissue injuries on their face, torso, and lower extremity, as these injuries displayed a high risk for NAT. Bruising on these areas in young children is much rarer in cases of accidental injuries. Skin and soft tissue injuries to the scalp and upper extremity were not statistically more likely to be seen in NAT than AT. Upper extremity injuries were seen in around the same percentage of children in NAT and AT cases, and the majority of the accidental cases it was seen in were the result of an MVC. Previous studies have found that bruises on the scalp are also rarely seen in accidental cases, so it does not by any means rule out NAT as a possibility [9]. Retinal injuries were found to be highly specific for NAT in this study. Retinal injuries have historically been a hallmark of NAT, often due to sheering forces experienced in abusive head trauma [5, 10, 11]. Only one case of accidental trauma from a fall resulted in a retinal injury in this study. Internal organ injuries should also raise significant suspicion for NAT. Organ injuries are less common than other injury patterns seen in NAT but are often seen in the more severe NAT cases and should warrant a higher index of suspicion during screening [12, 13]. The only two AT causes of internal organ injury in this study were caused by MVC.

When compared generally, the presence of a bone fracture was not statistically significant between cases of NAT and accidental trauma in infants. However, when analyzed by location of fracture, lower extremity fractures were highly associated with cases of NAT over accidental trauma. The rationale being that this population is unable to generate sufficient force to cause a significant self-inflicted injury [7, 14]. While skull fractures were not a significant indicator in NAT determination in this study, they were still present in over half of the cases and should still be considered a risk factor when evaluating an infant. Although it was not statistically significant, the presence of multiple fractures had a higher prevalence in NAT (46%) as compared to AT (27%) and should be concerning for potential NAT. The presence of multiple fractures in a young child is widely accepted to be indicators of abuse [7, 15].

Cerebral injuries were not significant for NAT overall or when broken down by type of injury to the brain. Cerebral injuries were seen in over half of the cases found to be accidental trauma, most commonly resulting from a fall. Cerebral injuries should still be flagged for suspicion of NAT because they were still present in 37.5% of the cases. In addition, abusive head trauma (AHT) has been shown in other studies to be one of the most common types of NAT and the most morbid and potentially fatal [4, 5, 10, 11].

The presence of multiple injury patterns was about equal by percent of cases for both NAT and AT. Although a child presenting to the ER with multiple injury patterns may in itself not be statistically higher risk for NAT than for accidental injury, it is still very important to screen any child with multiple injuries for NAT since over 2/3 of the cases of NAT did have multiple injury patterns.

Motor vehicle collisions (MVC) were the accidental cause that presented most similarly to NAT. MVC were the only accidental cause of injury that resulted in either torso, lower extremity, or internal organ injuries. Facial injuries were also seen in 87.5% of MVC. Facial injuries were also seen in 75% of victims of animal attacks and 9.1% of fall cases.

A significant finding of this study not previously mentioned elsewhere was that 1/3^rd^ of the NAT victims were discharged to the environment in which the trauma occurred. While the social services, legal authorities, and Child Protection Services in each state do their best to ensure that the perpetrator is removed from the child's life, the social circumstances of these victims remain the same, and this predisposes them to a higher risk of recurrent NAT. It is well known that the initial admission is a sentinel event, and a subsequent admission increases the risk of significant morbidity and mortality in NAT. It is important that trauma centers have a low threshold in screening infants for NAT on initial admission and readmitting children when there is any suspicion of harm. Medical evaluation and documentation should be particular and provide the evidence necessary for authorities to relocate victims to a safer environment if necessary.

## 5. Conclusion

NAT carries a high mortality risk for victims who could not be appropriately diagnosed on initial exposure. It is important to screen a large number of patients to ensure that nobody slips through the cracks and returns to an unsafe home. Limitations to this study include the sample size and single institution data. This study suggests that certain injury patterns carry a higher risk for NAT and corroborates a higher risk profile for Medicaid patients. Future research directives should further evaluate discharge disposition factors in NAT victims.

## Figures and Tables

**Table 1:** Comparison of nonaccidental and accidental trauma by demographic data.

	Nonaccidental trauma (*N* = 32)	Accidental trauma (*N* = 59)	*p* value
Age, *N* (%)			NS
Under 1 year	20 (62.5)	42 (71.2)	
1-2 years	12 (37.5)	17 (28.8)	
Sex, *N* (%)			NS
Male	21 (65.6)	33 (55.9)	
Female	11 (34.4)	26 (44.1)	
Race, *N* (%)			
Caucasian	13 (40.6)	25 (42.4)	NS
Hispanic/Latino	6 (18.8)	19 (32.2)	NS
Black	11 (34.4)	10 (16.9)	NS
Unknown	2 (6.25)	5 (8.5)	
Insurance, *N* (%)			
Medicaid	31 (96.9)	44 (74.6)	0.02^∗^
Private	1 (3.1)	9 (15.3)	
Dual coverage	0	4 (6.8)	
Missing data	0	2 (3.4)	

^∗^Represent significant data.

**Table 2:** Comparison of nonaccidental and accidental trauma by general injury pattern.

Injury pattern, *N* (%)	Nonaccidental trauma (*N* =32)	Accidental trauma (*N* =59)	*p* value
Facial	14 (43.8)	14 (23.7)	0.04^∗^
Scalp	11 (34.3)	27 (45.8)	NS
Upper extremity	5 (15.6)	9 (15.3)	NS
Torso	11 (34.4)	6 (10.2)	0.004^∗^
Lower extremity	7 (21.9)	3 (5.1)	0.01^∗^
Bone fracture(s)	13 (40.6)	33 (55.9)	NS
Cerebral	12 (37.5)	32 (54.2)	NS
Retinal	4 (12.5)	1 (1.7)	0.03^∗^
Internal organ	5 (15.6)	2 (3.4)	0.04^∗^
Multiple injury patterns	22 (68.8)	39 (66.1)	NS

^∗^Represent significant data.

**Table 3:** Comparison of fracture cases for NAT and AT.

Fracture type, *N* (%)	Nonaccidental trauma (*N* = 13)	Accidental trauma (*N* = 33)	*p* value
Skull	7 (53.8)	23 (69.7)	NS
Extremities	8 (61.5)	7 (21.2)	0.008^∗^
Ribs	2 (15.4)	1 (3.0)	NS
Multiple	6 (46.2)	9 (27.2)	NS

^∗^Represent significant data.

**Table 4:** Internal organ injury presentations in NAT and AT.

Internal organ injured, *N*	Nonaccidental trauma (*N* = 5)	Accidental trauma (*N* = 2)
Liver contusion/laceration	4	2
Cardiac arrest	2	0
Lung contusion	2	1
Pancreas contusion	0	1
Adrenal gland	1	0
Spleen capsular tear	1	0
Multiple organ injuries	3	2

**Table 5:** Comparison of cerebral injuries seen in cases of NAT and AT.

Cerebral injury, *N* (%)	Nonaccidental trauma (*N* = 12)	Accidental trauma (*N* = 32)
Subdural hematoma	9 (75)	15 (46.9)
Epidural hematoma	1 (8.3)	4 (12.5)
Intracerebral hematoma	3 (25)	3 (9.4)
Subarachnoid hemorrhage	1 (8.3)	3 (9.4)
Concussion or LOC	1 (8.3)	8 (25)
Seizure	2 (16.7)	1 (3.1)
Other	3 (25)	3 (9.4)
Multiple	3 (25)	3 (9.4)

^∗^Represent significant data.

## Data Availability

Data is not publicly available.
